# Illegitimate Tasks as an Impediment to Job Satisfaction and Intrinsic Motivation: Moderated Mediation Effects of Gender and Effort-Reward Imbalance

**DOI:** 10.3389/fpsyg.2016.01818

**Published:** 2016-11-21

**Authors:** Rachel Omansky, Erin M. Eatough, Marcus J. Fila

**Affiliations:** ^1^Baruch College and The Graduate Center, City University of New YorkNew York NY, USA; ^2^Hope CollegeHolland, MI, USA

**Keywords:** illegitimate tasks, well-being, effort-reward imbalance, gender differences, job satisfaction, intrinsic motivation

## Abstract

The current work examines a contemporary workplace stressor that has only recently been introduced into the literature: illegitimate tasks. Illegitimate tasks are work tasks that violate identity role norms about what can reasonably be expected from an employee in a given position. Although illegitimate tasks have been linked to employee well-being in past work, we know little about the potential explanatory mechanisms linking illegitimate tasks to work-relevant negative psychological states. Using a sample of 213 US-based employees of mixed occupations and a cross-sectional design, the present study examines job satisfaction and intrinsic motivation as outcomes of illegitimate tasks. Additionally, we examine perception of effort-reward imbalance (ERI) as a potential mediating mechanism through which illegitimate tasks relate to job satisfaction and intrinsic motivation, highlighting a possible pathway by which these relationships are functioning. Finally, we explore gender as a socially constructed variable that could contribute to variation in responses to illegitimate tasks and moderate the mediated link between illegitimate tasks and outcomes. Results indicated that illegitimate tasks were significantly related to job satisfaction and intrinsic motivation both directly and indirectly through perceptions of ERI in the predicted directions. Moreover, a moderated-mediation effect was found such that male workers reacted more than female workers to illegitimate tasks through the mechanism of perceived ERI.

## Introduction

Employee work well-being continues to be central to organizational research and practice, given its importance to individual workers, as well as to organizational and societal-level functioning (Griffin and Clarke, 2011). Two prominent indicators of work well-being are job satisfaction and intrinsic work motivation (see Aamodt, 2016). In addition to being important work-relevant psychological states, both are established predictors of various important individual and organizational-level outcomes. Job satisfaction has been linked to job performance (Spector, 1997), burnout (Faragher et al., 2005), and retention (Tett and Meyer, 1993). Similarly, intrinsic motivation has been positively linked to job performance (Ryan and Deci, 2000; Grant et al., 2007), as well as negatively related to turnover intentions (Richer et al., 2002), and burnout (Richer et al., 2002; Cerasoli et al., 2014). Given their importance to -and centrality within- work-related nomology, continued efforts are necessary to understand better how work conditions predict these outcomes. Furthermore, it is prudent to examine how occupational demands interact with prominent individual characteristics such as gender in predicting job satisfaction and intrinsic motivation because such characteristics tend to infiltrate the entire work experience (Lambert, 1991; Reed et al., 1994; Klassen and Chiu, 2010). The current work addresses both needs.

Stressful occupational demands have been repeatedly shown to undermine job satisfaction and intrinsic motivation (e.g., Spector, 1985, 1997; Rubino et al., 2009). The majority of existing research has focused on how high demand levels, as well as low levels of resources available to cope with demands, such as autonomy, decision making ability, and social support sources at work have produced decrements to these psychological states (e.g., Deci and Ryan, 1985; Dwyer and Ganster, 1991; Fila et al., 2014). However, given the changing nature of the workplace, it is imperative to examine contemporary understudied stressor demands that may have substantial explanatory power in the prediction of these outcomes *beyond* established forms of stress, in order to provide informed recommendations to leaders.

Illegitimate tasks (Semmer et al., 2005, 2007) represent one such contemporary work stressor that was recently introduced to the organizational psychology literature. Illegitimate tasks are assigned work demands that violate work role norms about what can reasonably be expected from an employee in a given position (Semmer et al., 2007), such as expecting a doctor to change a patient’s bedpan, which would usually be the job of a junior nurse. Other examples include requiring a legal secretary to meet with a client to discuss matters that should involve an attorney or conforming to strict office rules that don’t make any sense. Illegitimate tasks are not necessarily new to the workplace. However, the nature and structure of the evolving work environment, such as the US’s transition to more service-oriented industries and increasing individual perceptions that organizational citizenship behaviors are obligatory (Haworth and Levy, 2001), are such that illegitimate tasks are becoming a bigger issue for employees. Combined with the recent conceptualization and operationalization of the construct in the organizational psychology literature, we argue these factors merit the consideration of illegitimate tasks as a contemporary stressor.

Importantly, illegitimate tasks have been found to be distinct from and account for strain *beyond* the predictive ability of other more established work demand stressors (Semmer et al., 2010, 2015). In comparison to role overload (Karasek, 1979), for example, illegitimate tasks are not necessarily hard to complete but they violate the worker’s role identity based on the requirement to carry out demands that are out of context for the role, and the individual in question (Semmer et al., 2007, 2010). These tasks are also similar to, but conceptually distinct from, role conflict. Role conflict involves a conflict “between the focal person’s internal standards or values and the defined role behavior...” (Rizzo et al., 1970, p. 155), whereas illegitimate tasks refer to conflict between a task’s extrinsic qualities and one’s role expectations (Semmer et al., 2015). Illegitimate tasks have also previously been demonstrated to be distinct from justice constructs (i.e., distributive, procedural, and interactional justice) in that illegitimate tasks focus on fairness perceptions of task assignments while justice theories refer to fairness perceptions regarding allotment of positions, resources, and reward (Semmer et al., 2010, 2015). Indeed, illegitimate tasks predict outcomes above and beyond justice constructs (Semmer et al., 2015).

Extant research on this egregious stressor has primarily focused on associations between illegitimate tasks and various forms of psychological strain, as well as counterproductive work behavior (e.g., Kottwitz et al., 2013; Eatough et al., 2015–2016; Semmer et al., 2015). However, little attention has been paid to how illegitimate tasks might affect *positive* work-relevant job states such as job satisfaction or work motivation; with the exception of Eatough et al. (2015–2016), and Stocker et al. (2010), whose findings suggest illegitimate tasks reduce job satisfaction. Furthermore, few studies have explored the mechanisms underlying basic illegitimate tasks-outcome associations and none have examined gender as a potential moderator, despite much research showing gender differences in the interpretation of workplace stressors and subsequent coping (Roxburgh, 1996; Quick et al., 1997; Niedhammer et al., 1998). As such, we address these deficiencies by making three unique contributions to current understanding of illegitimate tasks and their relationships with employee work well-being. First, grounding our study in “Stress-As-Offense-to-Self” (SOS) theory (Semmer et al., 2007) and Warr’s (1990) model of well-being, we examine direct relationships between perceptions of task illegitimacy and both job satisfaction and intrinsic motivation, in order to establish illegitimate tasks as a negative correlate of these important markers of work well-being.

Second, we examine perceptions of effort-reward imbalance (ERI) as a possible mediator of these relationships. The ERI model (Siegrist, 1996) focuses on an individual’s efforts to achieve balance in the workplace through comparison of inputs and outcomes with others (Siegrist, 1996). In the following sections, we postulate that employees assigned tasks perceived as illegitimate will experience decrements to job satisfaction and intrinsic motivation because they perceive that the effort they must expend to complete such tasks is – by nature of their illegitimacy –imbalanced with the reward they will receive for doing so. We cannot gain a complete understanding of the effects of illegitimate tasks without moving to models that suggest explanatory links. The present study will elucidate whether ERI is a possible mechanism that explains why these tasks may be detrimental to desirable employee states.

Third, we examine whether illegitimate tasks are differentially damaging to work well-being based on employee gender. Employee gender continues to be at the forefront of organizational literature (e.g., Heilman and Eagly, 2008; Barreto et al., 2009), and it is now widely accepted by researchers that males and females perceive stress, and cope with stressors differently (Roxburgh, 1996; Quick et al., 1997; Niedhammer et al., 1998). Yet, we presently lack an understanding of how gender might influence task illegitimacy perceptions and, in turn, how these variables affect job satisfaction and intrinsic work motivation. Thus, we attempt to illuminate possible gender differences in illegitimate tasks perceptions, and explore how these perceptual differences relate to job satisfaction and intrinsic motivation in men versus women.

Taken together we aim to elucidate further how illegitimate tasks function in employees’ minds in relation to their job satisfaction, and their intrinsic motivation at work. In the following sections we outline the concept of illegitimate tasks in more detail, as well as review the possible mediating role of perceived ERI, and moderating role of gender on relationships with these positive work-relevant psychological states.

### Illegitimate Tasks

Illegitimate tasks can be conceptualized within the SOS framework (Semmer et al., 2007, 2015). SOS theory stems from the idea that individuals strive to maintain a positive self-view, which can be attained through personal self-esteem (i.e., positive self-evaluation; Epstein, 1998) or social esteem (i.e., positive evaluations by others; Leary and Baumeister, 2000). One factor that contributes to the development of one’s self-view is an individual’s work or professional role (Siegrist et al., 1986; Ashforth, 2001; Semmer et al., 2007). Work or professional roles become incorporated into an employee’s identity by providing a sense of meaning and purpose (Thoits, 1991). These roles are defined by normative prescriptions regarding what can and cannot be expected from occupants of a given work or professional role (Ilgen and Hollenbeck, 1991). Moreover, these normative prescriptions are usually shaped by more collective norms at the occupational and/or organizational levels (Semmer et al., 2005, 2010). As such, tasks or assignments that defy these norms may be perceived as *illegitimate* (Semmer et al., 2007, 2010).

Tasks may be perceived as illegitimate to the extent that they are viewed to be unreasonable or unnecessary. *Unreasonable tasks* are those considered to outside the boundaries of one’s occupational status, or one’s range of occupational abilities, such as assigning tasks to junior employees that require a greater level of skill, experience, or expertise than they can reasonably be expected to possess; or assigning tasks to more senior employees that demean their capabilities. Concomitantly, *unnecessary tasks* are those that employees believe no-one should have to perform, such as being required to complete meaningless paperwork, or being held to policies which make no sense (Björk et al., 2013). Tasks can also be perceived as unnecessary due to organizational inefficiencies, such as being required to enter identical data on two incompatible information technology systems (Semmer et al., 2015). Illegitimate tasks threaten one’s professional role identity by communicating disrespect, lack of appreciation, or negative evaluation by others (Semmer et al., 2007). Threats to professional role identity can threaten one’s positive self-view and self-esteem, which is stressful (Stets, 2005). Thus, illegitimate tasks are considered “identity-relevant stressors” (Thoits, 1991).

Although the concept of illegitimate tasks is still relatively new to organizational literature, research to date suggests that task illegitimacy has a myriad of negative implications for employee work well-being. Illegitimate tasks have been shown to correlate positively with employee stress (Björk et al., 2013), resentment both directly and indirectly via lack of appreciation (Stocker et al., 2010), and burnout (Semmer et al., 2010); and negatively with satisfaction with work performance (Björk et al., 2013), mental health (Madsen et al., 2014), and self-esteem (Semmer et al., 2010).

With regard to more complex study designs, daily illegitimate tasks predicted poorer sleep quality in Swiss employees (Pereira et al., 2014) and daily fluctuations in state self-esteem (Eatough et al., 2015–2016). Further, in a US sample, Eatough et al. (2015–2016) demonstrated that daily fluctuations in illegitimate tasks predicted evening anger and job satisfaction, and depressive mood. Finally, Kottwitz et al. (2013) found longitudinal evidence that employees’ who perceived illegitimate tasks and reported lower than average health had higher levels of cortisol, a biological indicator of stress. Illegitimate tasks also predicted irritability and resentment in Swiss employee’s 2 months later (Semmer et al., 2010).

### Warr’s Model of Well-Being

Warr’s (1990) model of well-being posits that affective work well-being can be conceptualized in terms of two independent dimensions: pleasure and arousal. *Pleasure* refers to the content of one’s feelings and is represented by a horizontal dimension whereas *arousal* refers to the degree of activation one is experiencing and is represented by a vertical dimension (Warr, 1999). These dimensions are mapped onto a circumplex of affective well-being with three main axes that serve as markers of well-being (Warr, 1990). The first axis solely represents pleasure and is measured using only the horizontal pleased-displeased dimension. The second and third axes involve both pleasure and arousal and run diagonally through the four quadrants. The second axis ranges from anxiety to contentment, with anxiety characterized as high arousal and low pleasure and contentment characterized as low arousal but high pleasure (Warr, 1990, 1999). The third axis ranges from depression (marked by low pleasure and arousal) to enthusiasm (marked by high pleasure and arousal). Individuals’ well-being is characterized based on their location on each axis (Warr, 1990, 1999).

Environmental factors, specifically job characteristics, are important predictors of employee work well-being, with various job aspects differentially predicting forms of well-being (Warr, 1990). As stated, illegitimate tasks have only been studied in relation to a limited set of aspects of well-being, with the majority of this work focusing on negative markers of well-being (e.g., resentment, anger, depression). Thus, the present study expands on existing work by examining two positively valenced markers of well-being (i.e., intrinsic motivation and job satisfaction), that are high and low arousal, respectively.

### Illegitimate Tasks, Job satisfaction, and Intrinsic Motivation

The nature and characteristics of illegitimate tasks suggest they will play a role in predicting positive markers of work well-being such as job satisfaction and intrinsic motivation. Job satisfaction is defined as a positive and pleasurable state resulting from an individual’s job appraisal or job experience (Locke, 1976). Job satisfaction is a dominant subject in organizational research due to its importance for individual and organizational health and well-being, and continued functioning, which has been well established (for reviews, see: Petty et al., 1984; Iaffaldano and Muchinsky, 1985; Loher et al., 1985; Blegen, 1993; Brown and Peterson, 1993; Judge et al., 2002; Faragher et al., 2005; Zangaro and Soeken, 2007), making it by far the most examined construct in organizational literature (Spector, 1997).

The few studies that have empirically examined the illegitimate tasks-job satisfaction link have found illegitimate tasks to be negatively related to job satisfaction (Stocker et al., 2010; Eatough et al., 2015–2016). There are several theoretical reasons as to why illegitimate tasks may be tied to job satisfaction. First, SOS framework describes that assigning employees illegitimate tasks poses a threat to employee social and self-esteem, which results in psychological damage and is stressful. Relatedly, employee self-esteem may be threatened because employees may appraise being assigned illegitimate tasks as signifying failure to achieve their goal of succeeding in their job (Semmer et al., 2007). This stress derived from employees’ inability to draw a positive sense of self from an important area of their identity will likely have negative implications for employee work well-being, for example, their job satisfaction (Semmer et al., 2007, 2010).

Second, job characteristics theory (Hackman and Oldham, 1975) states that task significance and task variety (i.e., the degree to which it is challenging) influence the meaningfulness of one’s job. As illegitimate tasks are perceived as unreasonable or unnecessary, they detract from significant or challenging work and will therefore reduce the perceived meaningfulness of one’s job. Reduced meaningfulness of one’s job will in turn negatively affect job satisfaction (Hackman and Oldham, 1975).

Third, illegitimate tasks may be particularly important in forming effort-reward perceptions as well, as explained in detail below. As stated, illegitimate tasks fall outside of one’s role expectations and may therefore offer little in regards to achieving one’s goals or affirming one’s work role identity (Semmer et al., 2007). Thus, individuals may perceive these tasks as creating a “reciprocity deficit” (van Vegchel et al., 2005) or imbalance by requiring effort but offering little reward.

Although the relationship between illegitimate tasks and intrinsic motivation has not yet been examined, theoretical evidence supports the negative relationship between illegitimate tasks and intrinsic motivation for several reasons. First, pride in professional roles assists us in maintaining a positive self-view (Stets, 2005). Social contexts that allow individuals to maintain positive self-views (Ryan and Deci, 2000) and that satisfy their need for competence facilitate the maintenance and enhancement of intrinsic motivation (Self-Determination Theory; Deci and Ryan, 1980, 1991). Examples of such supportive contextual events include getting positive feedback, desirable challenges, or intrinsically rewarding tasks (Richer et al., 2002), and not being subjected to negative evaluations (Ryan and Deci, 2000). Conversely, social contexts that thwart individuals’ ability to maintain positive self-concepts and feel competent are associated with poorer intrinsic motivation (Ryan and Deci, 2000). Illegitimate tasks may constitute one such unsupportive contextual event because they convey disrespect and negative social evaluation to those assigned these tasks.

Second, job characteristics theory (Hackman and Oldham, 1975) posits that jobs that are meaningful are linked to higher intrinsic motivation. As illegitimate tasks are perceived as peripheral to one’s job, may be discordant with one’s job role, or may be seen as unnecessary, they are unlikely to be perceived as meaningful (Hackman and Oldham, 1975). Thus, jobs that are wrought with illegitimate tasks are expected to degrade intrinsic motivation of the role occupant.

Third, because illegitimate tasks are outside of the core elements of an employee’s job, these tasks are not intrinsically rewarding (Semmer et al., 2007). Furthermore, as detailed below, these tasks may be perceived as unfair (Semmer et al., 2007) or as offering few reward for one’s efforts because they convey lack of appreciation, social devaluation, and are not instrumental in achieving desirable goals (van Vegchel et al., 2005; Semmer et al., 2007).

Taken together, we expect that employees who experience high levels of illegitimate tasks may suffer threats to identity, a lack of meaningfulness in their jobs, or a sense of resource over expenditure, and are therefore expected to report lower intrinsic motivation.

- Hypothesis 1: Perceptions of task illegitimacy will be negatively related to a) job satisfaction and b) intrinsic motivation.

### Effort-Reward Imbalance

The ERI model states that work characterized by high efforts (i.e., job demands or obligations) and low reward (i.e., money, esteem, and job security/career opportunities) creates perceptions of imbalance that result in employee strain reactions and impaired well-being (Siegrist, 1996; Siegrist, 2002). Employee’s assigned illegitimate tasks may perceive ERI because their efforts are not rewarded or these reward are insufficient. Indeed, Semmer et al. (2007) suggest that illegitimate tasks may be stressful because they provoke feelings of unfairness and resentment. Illegitimate tasks, by nature, may be perceived as offering little reward because they are not within-role performance duties for which the individual is getting paid, are often not intrinsically rewarding, and are not citizenship behaviors for which some relational value could be earned (Semmer et al., 2007). Consequently, illegitimate tasks are likely seen as poor opportunities, for which effort doesn’t lead to gain. Thus, assignment of illegitimate tasks is expected to positively predict employee perceptions of ERI.

- Hypothesis 2: Perceptions of task illegitimacy will be positively related to perceptions of ERI.

When employees expend effort and receive commensurate reward, it can signify to employees that they are successful and valued. However, a “reciprocity deficit” (van Vegchel et al., 2005) can signify devaluation, lack of respect and appreciation, unfairness, or negative evaluation (Semmer et al., 2007), which may threaten employees’ social esteem (Semmer et al., 2007) and positive self-concept (Siegrist et al., 1986). This threat can cause “stress as disrespect” (SAD; Semmer et al., 2007, p. 46), which can prompt strain reactions and negatively impact employee work well-being (Siegrist et al., 1986; Siegrist, 1996). For example, prior work has linked ERI perceptions to employee job dissatisfaction (de Jonge et al., 2000; van Vegchel et al., 2005).

Moreover, although there is a paucity of work examining the link between ERI and intrinsic motivation, a variety of motivation theories (e.g., equity theory, Adams, 1963; cognitive theory of emotion, Lazarus, 1991; expectancy theory of motivation and VIE theory, Vroom, 1964; Schönpflug and Batmann, 1989) purport that employees who perceive an imbalance between effort and reward will attempt to restore balance by either cognitively or behaviorally reducing their effort, or attempting to maximize their reward. Thus, employees may experience reduced intrinsic motivation regarding their work because (i) illegitimate tasks may not be tied to explicit reward within the job, and (ii) such tasks likely reduce the degree to which the work role fulfills esteem needs. Based on these theoretical and empirical arguments, we contend that because illegitimate tasks reduce or offer few reward, they will correlate with an increase in ERI perceptions, and in turn, predict reduced job satisfaction and intrinsic motivation.

- Hypothesis 3: Perception of ERI will be negatively related to a) job satisfaction and b) intrinsic motivation.
- Hypothesis 4: Perception of ERI will mediate the relationship between illegitimate tasks and a) job satisfaction and b) intrinsic motivation. Assignment of illegitimate tasks will be associated with higher ERI perceptions, which will be linked to decreased job satisfaction and intrinsic motivation.

### Gender as a Moderator

Finally, this study explores the moderating role of gender. Historically, women have been disadvantaged in the workplace, in part because of assumptions, perceptions, and stereotypes about their characteristics and capabilities (e.g., Heilman, 2001; Eagly and Karau, 2002; Lyness and Heilman, 2006). Although the outlook for women has improved with changing roles for both men and women, gender stereotypes are still pervasive and gender disparity issues are still relevant in today’s work environment. Gender role theory posits that the different social roles that men and women typically inhabit, and others’ expectations for conformity to these roles results in behavioral differences between men and women (Eagly, 1987). Specifically, females are believed to be more *communal* (concerned with the welfare of others), nurturing, and giving whereas men are believed to be more *agentic* or assertive, ambitious, and dominant (Eagly, 1987; Eagly et al., 2000).

Based on gender role theory (Eagly, 1987), men and women may perceive and respond differently to being assigned illegitimate tasks, partly due to underlying assumptions about gender roles. Because gender role theory outlines that social role norms include women being communal, giving, and less dominant, women may be both expected to and accustomed to carrying out illegitimate tasks more than their male counterparts. If so, women are expected to feel less threatened by illegitimate tasks as the experience is more closely aligned with socialized norms. Indeed, research suggests there are more expectations for women to perform such extra-role work and women are more likely to be penalized in performance evaluations if they do not (Allen and Rush, 1998).

Conversely, men may be more reactive to illegitimate task assignments because being subjected to demands characterized by unfairness or disrespect would be inconsistent with the dominant male gender role. Thus, being assigned demoting or demeaning work is inconsistent with this norm. To clarify: regardless of the recipient’s gender, illegitimate tasks are equally a violation of the recipient’s *professional* role. Yet, for men, such tasks may *also* violate gender roles, making illegitimate tasks a more salient identity threat to men than to women. Given theoretical and empirical evidence suggesting men and women behave consistently with their respective gender roles (e.g., Zanna and Pack, 1975; Christensen and Rosenthal, 1982; Eagly, 1987), men are expected to be more reactive to illegitimate tasks than women due to compounding role-violations (both professional and gender) inherent in the experience, which in turn is expected to predict stronger direct associations with lower job satisfaction and intrinsic motivation.

- Hypothesis 5a: Gender will moderate the direct relationship between illegitimate tasks and job satisfaction such that the link between illegitimate tasks and job satisfaction will be stronger for males than for females.
- Hypothesis 5b: Gender will moderate the direct relationship between illegitimate tasks and intrinsic motivation such that the link between illegitimate tasks and intrinsic motivation will be stronger for males than for females.

We also expect that gender may moderate these relationships indirectly through ERI perceptions. Because women are expected to be more caring and giving, they may have lower expectations for reward for performing this type of behavior. Further, because men and women are socialized differently and women have historically been disadvantaged in the workplace, women may have lower expectations from their jobs in terms of effort-reward links than men and women’s tolerance for undesirable task assignments may be higher (Lambert, 1991; Clark, 1997). Accordingly, Lambert (1991) found that women maintained higher levels of intrinsic motivation than men, despite reporting that their jobs were significantly less rewarding (i.e., lower in job autonomy, skill variety, and pay). Moreover, Clark (1997) found gender differences in job satisfaction ratings such that women were more satisfied than men, even after controlling for work values, personal characteristics (e.g., age, education) and job characteristics (e.g., pay, hours, occupation). A cross-national study by Kaiser (2007) suggests that this gender-job satisfaction paradox exists in multiple countries in which women face unequal or restricted labor market opportunities but does not exist in countries with modernized labor markets.

These findings support the notion that women have lower well-being expectations from work. Women may therefore be less reactive or threatened when faced with illegitimate tasks because they perceive less of an imbalance or perhaps even expect imbalance. Based on gender role theory and the empirical evidence outlined above, we predict a stronger positive link between illegitimate tasks and ERI for males than for females because these tasks are inconsistent with the male gender role and socialized expectations.

- H6a: Gender will moderate the indirect relationship between illegitimate tasks and job satisfaction via ERI, such that the indirect link will be stronger for males than for females.
- H6b: Gender will moderate the indirect relationship between illegitimate tasks and intrinsic motivation via ERI, such that the indirect link will be stronger for males than for females.

## Materials and Methods

### Design and Participants

Participants were 213 part-time and full-time employees in various predominantly junior-level positions, representing a 76% response rate. Advertisements for participants were placed on the regulated university research systems of three US higher education institutions and students who met criteria for the study expressed interest to the researchers. Participants ranged in age from 18 to 36 years, with a mean age of 20.9 years, and a standard deviation of 1.9 years. Forty-nine percent were women; 84.1% were Caucasian, 2.8% were African American, 9.3% were Asian, 1.9% were Hispanic or Latino, and the remaining 1.9% were of other ethnic groups (no participants were Native American). Participants averaged 18.6 hours of work per week, and had worked for their present organization for between 1 month and 12 years, with an average tenure of 1 year and 7 months. Participants provided consent prior to beginning the survey.

### Procedure

Surveys were administered through (i) an anonymous online data collection server, or (ii) an anonymous paper and pencil survey, depending on system for data collected approved at the institution where the data were collected. All surveys were administered at a single time point. Participation was voluntary, and all participants were assured of both identity and response confidentiality as participants was anonymous within the dataset. Course credit was offered as compensation for participation. Institutional review board approval was obtained to complete this study.

### Measures

#### Illegitimate Tasks

Illegitimate tasks were assessed using the eight-item Bern Illegitimate Task Scale (Semmer et al., 2015). Sample items included: “Do you have work tasks to take care of, which you believe should be done by someone else?” (unreasonable tasks), and “Do you have work tasks to take care of, which keep you wondering if they have to be done at all?” (unnecessary tasks). Participants rated each item on a 5-point Likert scale (1 = strongly disagree; 5 = strongly agree). The reliability coefficient for the scores for this sample was 0.88.

#### Effort-Reward Imbalance Perceptions

van Yperen’s (1996) six-item, 5-point scale was used to examine perceptions of ERI (1 = strongly disagree; 5 = strongly agree). A sample item is, “I invest more in my job than I receive in return,” (*α* = 0.89).

#### Intrinsic Motivation

Gagné et al.’s (2010) three-item 5-point Motivation at Work scale (MAWS) was used to assess intrinsic motivation (1 = strongly disagree; 5 = strongly agree). An example item includes, “I feel motivated because I enjoy this work very much,” (*α* = 0.92).

#### Job Satisfaction

To assess job satisfaction, Cammann et al. (1979, unpublished) three-item 5-point scale was used (1 = strongly disagree; 5 = strongly agree). A sample item is, “All in all, I am satisfied with my job,” (*α* = 0.90).

#### Demographics

Demographic information was collected from participants including age, gender, and ethnicity.

### Data Analyses

Using SPSS 21 statistical software, Hypotheses 1–3 were tested with bivariate Pearson correlations. Hypotheses 4, 5, and 6 were tested using moderated mediation analyses. The SPSS PROCESS macro software by Hayes (2013) was used to conduct these analyses. Model 8 was used to test Hypotheses 4–6.

## Results

### Main Effects

Bivariate correlations were used to test Hypotheses 1–3 and are reported in **Table 1**. Our results indicated that illegitimate tasks was significantly negatively related to job satisfaction (*r* = –0.41, *p* < 0.01) and intrinsic motivation (*r* = –0.25, *p* < 0.01), in support of hypotheses 1a and 1b. Illegitimate tasks was positively linked to ERI (*r* = 0.52, *p* < 0.01), in support of Hypothesis 2. Moreover, ERI perceptions were negatively related to both job satisfaction and intrinsic motivation (*r* = –0.49, *p* < 0.01, and *r* = –0.28, *p* < 0.01, respectively), thus supporting hypothesis 3a and 3b.

**Table 1:** Means, standard deviations, and correlations of study variables.

			Item						
Item	Mean	*SD*	1	2	3	4	5	6	7
(1) Age (Years)	20.89	1.93	–						
(2) Tenure (Years)	1.62	1.73	0.34^∗∗^	–					
(3) IT^a^	2.75	0.79	0.07	0.12	(0.88)				
(4) ERI^b^	2.63	0.88	0.12	0.15^∗^	0.52^∗∗^	(0.89)			
(5) Gender^c^	1.49	0.50	–0.23^∗∗^	–0.09	0.00	–0.02	–		
(6) Job satisfaction	3.55	1.04	–0.01	–0.05	–0.41^∗∗^	–0.49^∗∗^	0.10	(0.90)	
(7) Intrinsic motivation	3.33	1.08	0.05	–0.03	–0.25^∗∗^	–0.28^∗∗^	0.06	0.71^∗∗^	(0.92)

*N = 208–214.*^a^*IT, Illegitimate tasks.*^b^*ERI, effort-reward imbalance.*^c^*1 = Male, 2 = Female. Coefficient alphas are on the diagonals and correlations are below diagonals.*^∗^*p < 0.05, ^∗∗^p < 0.01.*

### Moderated Mediation Analyses

Results of moderated mediation analyses for job satisfaction and intrinsic motivation are illustrated in **Tables 2** and **3**, respectively. Hypothesis 4 stated that ERI perceptions would mediate the relationship between illegitimate tasks and both job satisfaction and intrinsic motivation, respectively. Perceptions of ERI mediated the illegitimate tasks-job satisfaction link (0.12, 95% CI 0.02, 0.24) and the illegitimate tasks-intrinsic motivation link (0.07, 95% CI 0.01, 0.17), with the bootstrapped confidence intervals around both indirect effects not containing zero. Thus, support for hypotheses 4a and 4b was found.

**Table 2:** Results of test of moderated mediation with job satisfaction as outcome.

	Job satisfaction				ERI			
Predictor	*B*	*SE*	LLCI	ULCI	*B*	*SE*	LLCI	ULCI
Illegitimate tasks	–0.31	0.25	–0.79	0.18	1.04^∗∗∗^	0.21	0.64	1.45
Gender	0.17	0.40	–0.66	1.0	0.80^∗^	0.37	0.07	1.53
ERI	–0.39^∗∗∗^	0.08	–0.54	–0.24				
Illegitimate tasks × Gender	0.00	0.15	–0.29	0.30	–0.30^∗^	0.13	–0.56	–0.05
*R^2^*	0.10^∗∗∗^							

**Indirect effects**								
	***B (SE)***				**95% CI**			
	
Illegitimate tasks	0.12 (0.05)				[0.02, 0.24]			
Illegitimate tasks × Gender	0.12 (0.05)				[0.02, 0.24]			

*ERI, effort-reward imbalance. Bs represent unstandardized coefficients. SE, standard error. LLCI, lower level confidence interval. ULCI, upper level confidence interval. ^∗^p ≤ 0.05, ^∗∗∗^p ≤ 0.001.*

**Table 3:** Results of test of moderated mediation with intrinsic motivation as outcome.

	Intrinsic motivation				ERI			
Predictor	*B*	*SE*	LLCI	ULCI	*B*	*SE*	LLCI	ULCI
Illegitimate tasks	–0.14	0.30	–0.73	0.45	1.04^∗∗∗^	0.21	0.64	1.45
Gender	0.27	0.51	–0.74	1.28	0.80^∗^	0.37	0.07	1.53
ERI	–0.22^∗^	0.09	–0.41	–0.03				
Illegitimate tasks × Gender	–0.06	0.18	–0.41	0.30	–0.30^∗^	0.13	–0.56	–0.05
*R^2^*	0.30^∗∗∗^							

**Indirect effects**								

	***B* (*SE*)**				**95% CI**			
	
Illegitimate tasks	0.07 (0.04)				[0.01, 0.17]			
Illegitimate tasks × Gender	0.07 (0.04)				[0.01, 0.17]			

*ERI, effort-reward imbalance. Bs represent unstandardized coefficients. SE, standard error. LLCI, lower level confidence interval. ULCI, upper level confidence interval. ^∗^p ≤ 0.05, ^∗∗∗^p ≤ 0.001.*

**FIGURE 1:**
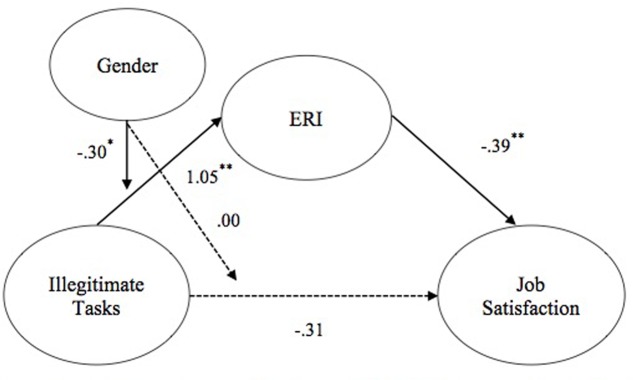
**Tested moderated mediation model for illegitimate tasks and job satisfaction.** Solid paths represent significant links found. Dashed paths represent links that were hypothesized but non-significant. Parameters represent unstandardized beta coefficients, ^∗^*p <* 0.05, ^∗∗^*p <* 0.01.

The results of our conditional mediation analyses were mixed. First, hypotheses 5a and 6a proposed that gender would moderate the direct and indirect relationships between illegitimate tasks and job satisfaction, such that both the direct and indirect relationships would be stronger for males than for females. Results indicated that gender did not moderate the direct path between illegitimate tasks and job satisfaction (0.00, *ns*), thus failing to support Hypothesis 5a. However, Hypothesis 6a was supported because illegitimate tasks predicted greater ERI perceptions (1.04, *p* < 0.01) and ERI perceptions, in turn predicted lower levels of job satisfaction (-0.39, *p* < 0.001). Additionally, gender moderated the mediated pathway (-0.31, *p* < 0.05), with the bootstrapped confidence interval around the index of moderated mediation not including zero (0.11, 95% CI 0.02, –0.24), such that males were more reactive to illegitimate tasks (-0.29) than were females (-0.17). See **Figure 1**.

**FIGURE 2:**
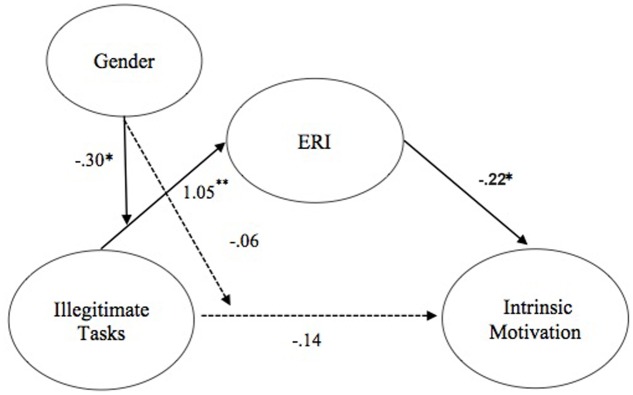
**Tested moderated mediation model for illegitimate tasks and intrinsic motivation.** Solid paths represent significant links found. Dashed paths represent links that were hypothesized but non-significant. Parameters represent unstandardized beta coefficients, ^∗^*p <* 0.05, ^∗∗^*p <* 0.01.

Second, hypotheses 5b and 6b stated that gender would moderate the direct (5b) and indirect relationship (6b) between illegitimate tasks and intrinsic motivation such that these links were stronger for males than for females. Gender did not moderate the direct path (0.06, *ns*), thus failing to support hypothesis 5b. However, support for hypothesis 6b was found because illegitimate tasks predicated greater ERI perceptions (1.04, *p* < 0.05), which in turn predicted lower intrinsic motivation (-0.22, *p* < 0.05). In addition, gender moderated the mediated pathway (-0.31, *p* < 0.05), with the bootstrapped confidence interval around the index of moderated mediation not including zero (0.06, 95% CI 0.01, –0.07) such that males demonstrated a stronger link between illegitimate tasks and intrinsic motivation via perceptions of ERI (-0.16) than females (-0.10). See **Figure 2**.

## Discussion

The goal of this study was to broaden knowledge of how illegitimate tasks may negatively relate to job satisfaction and intrinsic motivation. Illegitimate tasks are thought to impact strain and well-being based on social threats to identity (Semmer et al., 2007, 2015). However, most research to date on illegitimate tasks has focused on psychological strain and negative behavioral outcomes (e.g., Semmer et al., 2010, 2015). Thus, in accordance with SOS theory and Warr’s (1990) model of well-being, we aimed to show that perceptions of task illegitimacy may also diminish forms of work well-being – that is, job satisfaction and intrinsic motivation – that have been repeatedly shown to affect both personal and organizational-level functioning. Additionally, few studies have explored the mechanisms underlying basic illegitimate tasks-outcome associations. Thus, we also explored ERI as a possible mediator of these relationships, given clear theoretical links between perceived task illegitimacy and unfairness, and the likelihood of employees perceiving reciprocity deficits (van Vegchel et al., 2005) if tasks are perceived to be illegitimate. Finally, in accordance with gender role theory (Eagly, 1987), and extensive research on the role of gender in appraising, and coping with stress, as well as ongoing gender role norms in the workplace, we examined gender as a moderator of this mediated relationship.

Our results for hypothesis 1 suggest that illegitimate tasks are negatively related to job satisfaction and intrinsic motivation. This is consistent with SOS theory (Semmer et al., 2007), which suggests illegitimate tasks can create psychological damage; and with prior work demonstrating the importance of justice perceptions for employee motivation (Deci and Ryan, 2000; Colquitt et al., 2001; Zapata-Phelan et al., 2009). These results also expand our knowledge about the scope of illegitimate tasks, as they suggest that in addition to links with psychological strain and negative behavioral outcomes found in previous studies (e.g., Semmer et al., 2010, 2015), there are possible implications of perceived task illegitimacy for *positive* forms of work well-being, in accordance with Warr’s (1990) model. If so, managers and job design consultants are advised to ensure that task assignments align with identity role norms about what can reasonably be expected from an employee in a given position (Semmer et al., 2007), or else employees may lose the meaningfulness of their job (Hackman and Oldham, 1975), fail to achieve their goals, or compromise their work role identity (Semmer et al., 2007).

Second, our results also support the plausibility that perceived ERI fully mediates the path between illegitimate tasks and both job satisfaction and intrinsic motivation. This finding is in line with past work that has suggested that inability to maintain a positive self-view or expending effort with little reward is problematic for well-being (Hackman and Oldham, 1975; Deci and Ryan, 2000). In addition, this finding supports the idea that employees may disengage from their role or lose drive to perform within professional roles if given tasks that undermine the appropriate role boundary. This may be damaging to organization when managers believe they have achieved an appropriate balance between effort demanded and reward afforded to employees and yet, if tasks are thought to be illegitimate by employees, then they may perceive ERI, even if the overall workload appears to be commensurate with reward. Thus, our finding offers new context to previous knowledge of ERI, because it suggests that the *legitimacy* of tasks within the workload (e.g., the *effort*) must be considered in addition to the amount of work assigned. Otherwise, managers might erroneously presume that balance between effort and reward has been achieved, and may be unprepared for the consequences (e.g., Siegrist, 1996, 2002).

Finally, our findings illustrate that reactions to illegitimate tasks, while pervasive, may vary based on gender. Specifically, gender moderated the indirect relationship between illegitimate tasks and job satisfaction and intrinsic motivation through ERI such that males were, on average, more reactive to illegitimate tasks than females. These findings build upon previous research on gender differences in interpretation of stressors and coping with stressors (Day and Livingstone, 2003) as well as with the notions of gender role theory (Eagly, 1987) that socialized gender norms (e.g., females as more communal, and males as more agentic) are likely to influence these reactions.

Our findings regarding the role of gender in interpreting and dealing with illegitimate tasks raise several important points for consideration. For example, despite widespread movement toward gender equality at work, might the disadvantaged history of women and the continued existence of gender disparity issues promote a pressure that may be real -or perceived- for women to adhere to illegitimate tasks – more so than for men? If so, this may explain why women displayed weaker links between illegitimate tasks and outcomes. Perhaps they are expecting such treatment or are less threatened by such requests. Furthermore, to the extent that illegitimate tasks both directly, and indirectly through a mediating mechanism of ERI, negatively relate to job satisfaction and intrinsic motivation, the performance of women may suffer more greatly than men if they are more readily expected to (or prepared to) acquiesce to illegitimate tasks than their male counterparts. Clearly, more research is needed to understand better the effects of gender on the interpretation of, and adherence to illegitimate tasks. However, one possible implication is that critical progress toward gender equality at work might be being undermined by tolerance of this stressor. For example, if women’s performance levels were to suffer more greatly, on average, than those of men, women’s progress in organizational hierarchies are likely to be inhibited with perhaps lasting implications for their upward mobility. An even more ominous view is that this truncation of progress might not be viewed by an observer as gender-biased. In other words, this problem could be quite subtle and difficult for organizations to identify, especially because it is unlikely that objective performance measurements indicate willingness or readiness to absorb illegitimate tasks and how this may have cascading consequences for overall effectiveness.

It is also important to consider the negative implications of illegitimate tasks for male workers. That our findings show males to be more likely than females to react to illegitimate tasks has thus far been discussed as a negative for women. However, greater decrements to job satisfaction and intrinsic motivation are, on the basis of other established relationships in the literature, more likely to result in male workers’ performance suffering than female workers. Consequently, males may be more likely to withdraw from their organization. An obvious concern with this possibility is that organizations may lose quality male employees who believe they are being asked to complete task assignment that they shouldn’t be. However, in contrast to promoting greater levels of gender equality at work, this potential state of events may essentially lower the bar for women by reducing the satisfaction and intrinsic motivation of male workers such that performance may suffer and males may be more inclined to leave. Critically, this would give no credence to the ability of female employees to continue progressing in the manner they have been over recent decades. Clearly then, illegitimate tasks may have negative consequences for both genders, with harmful implications for not only the individuals themselves, but organizational- and societal-level functioning. We therefore invite and encourage future research on this subject, which may shed an entirely new light on respective gender roles in the workplace.

### Limitations

We acknowledge several limitations of our study. First, the present study utilized a correlational research design, which constrains us from drawing conclusions about causality. Further, our analyses were cross-sectional, which limits inferences. Cross-sectional studies have been widely criticized in organizational literature for allowing reverse- or reciprocal-causation (e.g., Spector and Brannick, 1995; Spector, 2006). Moreover, cross-sectional design does not allow for the examination of whether repeated exposure to illegitimate tasks has an exacerbated or additive effect on ERI, job satisfaction, and intrinsic motivation. Replication of this study using a longitudinal or experimental design is necessary to clarify the directionality of these relationships, to fully support ERI as a mediator, and to explore the potential effect of time on these relationships. Third, the variables in this study were all assessed using self-report measurement, which raises the concern of whether common method bias could have impacted our results. According to Spector (2006), common method variance can be overstated, and study design should be based primarily on its purpose and the researcher’s desired inference. However, future work may consider alternative sources for reports. For example, an experimental or quasi-experimental study of a sample of employees in a field with well-defined tasks could manipulate the legitimacy of assigned tasks. Similarly, balance between effort and reward could be manipulated (e.g., Siegrist et al., 2005).

Additionally, the present study examined a relatively limited set of outcomes. Future studies should expand on the present work by exploring additional outcomes outside of the realm of work well-being including behaviors, perceptions, and other attitudes. In particular, researchers should consider possible cross-domain effects if the stress resulting from illegitimate task assignments spills over into non-work domains. For example, Lyness and Erkovan (2016) recently addressed career constructs that have not always been well represented in the work-family literature. Examining the role of the role of illegitimate tasks across the work-family domain would be a timely next step in understanding this egregious stressor. Finally, while the present study found support for gender as mediated moderator of the proposed relationships, it is possible that gender is merely a proxy for socially constructed differences in values, perceptions, personality characteristics, or behavioral tendencies. Future studies should examine specific attitudinal or behavioral characteristics, such as degree to which individuals display communal versus agentic self-view (e.g., Eagly et al., 2000), to determine whether such variables explain some or all of the variance accounted for by gender.

## Conclusion

Consistent with SOS theory (Semmer et al., 2007), the current study suggests the assignment of illegitimate tasks may threaten one’s job satisfaction and intrinsic motivation by creating the perception of an ERI. The present study also found that gender predicted the strength of the relationship between illegitimate tasks and outcomes such that men demonstrated stronger ties to negative outcomes than did women. As suggested by gender role theory (Eagly, 1987) and SOS theory (Semmer et al., 2007), illegitimate tasks should violate men’s professional role and, additionally, may threaten their gender role as they signal lack of consideration, thought, and respect from others, which is inconsistent with an agentic self-view.

The present study contributes to the expanding literature on illegitimate tasks by adding to the construct’s nomological network and exploring the processes by which illegitimate tasks may be negatively related to desirable employee attitudes and motivational states. This study found support for ERI as a mechanism that might explain this linkage. This work has also identified gender as a potentially important moderator of the relationships between illegitimate tasks and work-relevant outcomes: gender. This work was a first step into examining both why these tasks may relate to lower job satisfaction and intrinsic motivation and gender’s role in the process.

## Author Contributions

RO: Co-wrote the manuscript draft and revised based on co-author comments. Put together materials for submission. EME: Generated initial idea, submitted IRB, collected and analyzed data, co-wrote manuscript. MJF: Generated initial idea, submitted IRB, collected and analyzed data, co-wrote manuscript.

## Conflict of Interest Statement

The authors declare that the research was conducted in the absence of any commercial or financial relationships that could be construed as a potential conflict of interest.
